# Network Structure and Travel Time Perception

**DOI:** 10.1371/journal.pone.0077718

**Published:** 2013-10-29

**Authors:** Pavithra Parthasarathi, David Levinson, Hartwig Hochmair

**Affiliations:** 1 CDM Smith, New Haven, Connecticut, United States of America; 2 Department of Civil Engineering, University of Minnesota, Minneapolis, Minnesota, United States of America; 3 Fort Lauderdale Research and Education Center (FLREC), University of Florida, Fort Lauderdale, Florida, United States of America; University of Gävle, Sweden

## Abstract

The purpose of this research is to test the systematic variation in the perception of travel time among travelers and relate the variation to the underlying street network structure. Travel survey data from the Twin Cities metropolitan area (which includes the cities of Minneapolis and St. Paul) is used for the analysis. Travelers are classified into two groups based on the ratio of perceived and estimated commute travel time. The measures of network structure are estimated using the street network along the identified commute route. T-test comparisons are conducted to identify statistically significant differences in estimated network measures between the two traveler groups. The combined effect of these estimated network measures on travel time is then analyzed using regression models. The results from the t-test and regression analyses confirm the influence of the underlying network structure on the perception of travel time.

## Introduction

Travel demand forecasts are historically inaccurate [1]. There are many reasons for this, but one explanation is that the models embedded in the forecasts do not reflect actual behavior of individuals, or correctly characterize their perceptions. In particular, models assume individuals estimate travel times as accurately and consistently as the models themselves when making decisions (i.e. the estimated true travel time is an input into choice of destination, mode, and route). However, travelers typically misestimate travel times between locations, even frequently traveled locations like home and work, which may explain why they don’t choose the observed shortest travel time path [2]. This paper tests whether those misestimations can be systematically explained by network structure.

The perception or cognition of distance and travel time has a rich history in behavioral psychology and spatial geography. Geographers have focused their efforts in understanding the role of spatial patterns in influencing distance or travel time cognition. The general layout and topography of a city provides an inherent legibility [3]. Cities with a formal structure (e.g. London) characterized by crucial structural elements such as a river, rail network or road network have less over-estimation in distance than cities without a formal structure (e.g. Edinburgh). In a recent experiment, participants estimated a walk in a picturesque village to be twice as long as an equal-length journey in the city [4]. The analysis suggested that an individual’s scale of interaction with the environment influences the judgement of distance.

The factors that affect distance cognition are categorized as [5], [6]:

1. *stimulus-centered factors*, in which cognitive distance is a function of environmental features;
2. *subject-centered factors*, in which cognitive distance is a function of the individual;
3. *subject/stimulus-centered factors*, in which cognitive distance is a function of interactions between the individual and environmental features.

A similar listing of factors that influence an individual’s estimate of distance within an urban context was provided by [7].

Routes where the change in direction is enforced more often, e.g. turns, are perceived to be longer [8]. Two structural attributes along a route, namely angularity effects (right angle turns) and intersections, increased the perception of traversed distance [9]. These findings were consistent in both laboratory and field settings, using virtual environmental tools [10]. Distance towards the city center was estimated to be shorter than distances away from the city center [11]. Other attributes shown to affect perceived distance include information along the route (e.g. number of perceived features, such as buildings) or the visibility of the destination [12].

Perception of length varies by sex and age [13]. For men, the number of available routes had no effect on the accuracy of route length estimates, while the accuracy of length estimates decreased for women with exposure to multiple routes [14]. There were no significant differences in the estimation of straight-line distance between taxi drivers and the general public, however taxi drivers consistently estimated travel distances to be shorter than the general public [15].

The research focus in the 1970s was mainly on the cognition of physical distance between points, typically home and other locations within the city. The focus on time perception started later with an understanding that travel time is much more important for travelers than actual physical distance [6], [16]. Time perception has deep roots in psychological research due to time being an important aspect of human experience. An individual’s notion of time applies to two unique concepts: Succession and Duration. Perception of duration and succession is present very early in life but the joint functioning of the two concepts is not acquired until the age of 7 or 8 when the child is capable of logical thinking [17].

Time perception is now receiving attention in the travel behavior research. It is known to vary for transit by elements of the trips [18] and for highways by type of driving (e.g. stopped at a ramp meter or traffic signal, free-flow, or stop-and-go conditions) [19], [20]. The results from a Bayesian analysis of the role of travel time perception in route choice indicated that the individual’s perception of travel times strongly influenced the convergence of the traffic system [21]. The accuracy of car drivers’ perception of public transport travel time affects mode choice [22]. Researchers have also highlighted the limitations of using observed attribute values rather than perceived values in estimating the utility function in mode choice models using simulation experiments [23].

This paper extends the current research interest to understand the role of network structure in influencing an individual’s perception of travel time. The basic question addressed in this paper is:

*Do travelers perceive travel time differently and can the differences in perceived travel times be attributed to network structure?*

Network structure is a measure of the layout, arrangement and connectivity of the network including characteristics of the individual elements [24], [25]. Transportation geographers in the 1960s viewed transportation network structure as a vital input to understanding a region’s spatial patterns and economic development. Graph theory based approaches were used to quantify and analyze the underlying network structure and topology [26]–[29]. However these approaches were limited by data availability and computing power [30], [31].

The interest in understanding the topological properties of networks has a rich history in a variety of fields including mathematics, physics, biology and sociology [32]. For example, sociologists studied the structure and complexity of social networks to explain the social phenomena in a wide variety of disciplines from psychology to economics [33]. Similarly computer scientists used the measures of network structure developed by Kansky [26] to understand the complexity and reliability of computer networks. Physicists and mathematicians analyzed network structure to better understand the small-world properties of complex networks.

Recent advances in spatial techniques and associated improvements in computing power have brought about a renewal of interest in understanding complex systems such as the internet, social networks, biological networks, and transportation networks [34]. These advances in spatial techniques have allowed researchers to move away from the traditional representation of complex networks to developing meaningful representations that help uncover the underlying topological patterns. Refer to [35] for a detailed review of the progress in the modeling and analysis of complex networks.

Many of these new approaches have their roots in space syntax. Space syntax is an urban analysis technique originally developed to understand the pedestrian mobility patterns in cities, which has since been extended to understand other aspects such as modeling urban traffic, crime mapping, prediction of air pollution levels, etc. [36]. These approaches are considered to provide a better understanding of human perception and cognition [37], [38].

The current focus on complex systems is geared towards analyzing not just the underlying topology but their spatial or geographical aspects as well. This shift is mainly due to the understanding that the topology of a network is actually correlated to the underlying spatial structure [31]. For example, in road networks, the number of links or road segments connected to a single node or intersection is constrained by the available space. Similarly the number of connections in the airline networks is a function of the available space at the airports. Hence a complete understanding of network structure needs to consider both the spatial and topological aspects [39].

The understanding of network structure has applications in numerous fields ranging from urban planning to epidemiology [31]. Applications include studying the temporal evolution and growth of transportation networks [40]–[42]; comparing system performance [43]; understanding the formation of urban structure [44]; analyzing knowledge transfer in informal networks [45]; predicting the patterns of global epidemics [46]; comparing the organization of human brain networks between patient groups [47].

The role of network structure in influencing travelers’ perception of travel time is analyzed in this paper. The next section outlines the data used in the analysis. This is followed by a discussion of measures of network structure, data analysis, and the presentation of results.

## Methods

### Travel Survey and Network Data

The data for the analysis come from the Twin Cities metropolitan area. The Twin Cities metropolitan area refers to the seven counties of Anoka, Carver, Dakota, Hennepin, Ramsey, Scott and Washington and includes the cities of Minneapolis and Saint Paul.

#### Dataset I - Travel behavior inventory

The travel data for this analysis come from two different sources. The first dataset is the year 2000 Twin Cities Travel Behavior Inventory (TBI). The TBI is a comprehensive one-day household travel survey conducted by the Metropolitan Council and the Minnesota Department of Transportation (Mn/DOT). Survey respondents maintain a complete record of all trips undertaken on the specified travel day. In addition respondents also provide relevant individual and household-level socio-demographic data (ex. age, gender, household size) [48].

The surveyed sample includes households in the seven counties within the Twin Cities metropolitan area and twelve adjacent counties. The final sample consists of 6,219 households comprising 14,671 individuals, totaling 58,345 trips. The data are extracted to include only those trips that originated from and were destined for the Twin Cities metropolitan area resulting in 38,432 trips. The data on commute trips and reported trip arrival and departure times are most relevant to this analysis.

The dataset of 38,432 trips has been cleaned to remove records with missing or unreasonable attribute values. Table 1 summarizes the exclusion rules used to obtain the final dataset. The final dataset used in this analysis consists of 4,050 records.

**Table 1:** Data exclusion rules - TBI dataset.

	No. of observations
Number of initial records in the TBI dataset	38,432
Excluding non-commute trips	32,298
Excluding records using non-auto modes	354
Excluding duplicate records (i.e. trips with the same origin and destination)	1,067
Excluding records with missing data	566
Excluding records with missing or unreasonable network attributes	46
Excluding trips with unreasonable reported times	12
Excluding records with unreasonable tau values	39
**Number of records used in the analysis**	**4,050**

#### Dataset II - Surveys from the I-35W bridge collapse and reopening

The second dataset comes from a compilation of surveys conducted during the collapse and subsequent rebuilding of the Interstate 35W Highway Bridge over the Mississippi River in Minneapolis. The I-35W bridge collapsed on August 1, 2007 and the newly reconstructed bridge was open to traffic on September 17, 2008. The surveys were conducted as part of a research effort at the University of Minnesota to understand the impacts of the bridge collapse on traveler behavior [49]. The collected surveys are listed below:

#### P-2007

A hand-out/mail-back paper survey was conducted in September 2007, immediately after the collapse of the I-35W bridge. This was the first survey conducted as part of the research effort mentioned above. This survey focused mainly on two communities closest to the I-35W bridge and thus significantly affected by the bridge collapse: the downtown area of the City of Minneapolis and the Minneapolis campus of the University of Minnesota. Consistent with prior research this survey is denoted as P-2007.

Respondents were asked to provide relevant information on home and work locations, commute trip arrival and departure time, travel mode and socio-demographics. In addition, respondents were asked to draw their actual commute routes on street maps provided for this purpose. This information was collected for four phases, namely:

- Phase 1 - Before the bridge collapse (e.g., in July 2007),
- Phase 2 - The second day after the bridge collapse (e.g., August 2, 2007),
- Phase 3 - The following weeks (e.g., Aug. 3 to Aug. 30, 2007),
- Phase 4 - Current status (at the time of the survey).

A total of 1000 surveys were handed out and 141 completed surveys were received. The analysis in this paper uses auto based trips from Phase 4 of this survey.

#### W-2007

A computer-based internet survey was conducted in September 2007 and administered in eight zip codes in the Twin Cities area. Consistent with prior research, this survey is denoted W-2007.

A detailed description of the data collection efforts is provided in [50]. A recruitment postcard for the online survey was sent out to a pool of 5,000 individuals. The completed dataset consisted of 215 surveys, of which 167 surveys were usable. The final dataset of auto based trips, used in this analysis, consisted of 136 records. The survey explicitly asked respondents to provide their home and work locations along with an estimate of their travel time for the commute trip but it did not ask participants to provide their actual commute routes.

#### P-2008

A hand-out/mail-back paper survey was conducted in October 2008, immediately after the opening of the I-35W replacement bridge. Consistent with prior research, this survey is denoted P-2008. This survey is an extension of the P-2007 survey and is similar in terms of the methodology and focused on the same two communities. A total of 840 surveys were handed out and 137 completed surveys were received. The information on commute trips was collected for the following five phases:

- Phase 1 - Before the bridge collapse (e.g., in July 2007),
- Phase 2 - Before the bridge reopening (e.g., September 17, 2008),
- Phase 3 - After the bridge reopening (September 18, 2008),
- Phase 4 - The following weeks (Sept. 19 to Oct. 23, 2008),
- Phase 5 - Current status (at the time of the survey).

The analysis in this paper uses auto based trips from Phase 4 of the survey.

#### G-2008

Global positioning systems (GPS) were installed in subject vehicles and GPS data were collected as part of several other projects for a time period of thirteen weeks, three weeks before the reopening of the I-35 W replacement bridge on September 17, 2008 and between eight to ten weeks after that. Logging GPS units were installed in subject vehicles and accurately monitored the travel trajectories of the vehicle at a frequency of one point every 25 meters [49].

The data downloaded from the GPS units provide information on the trip origin and destination along with the actual routes and trip travel times. The information on home and work locations and reported commute travel time had been previously obtained as part of the recruitment questionnaire. This information on home and work locations was used to extract the commute trips from the GPS dataset. The GPS units were installed in 127 vehicles and the final dataset used here consisted of 72 usable units with complete information. To ensure consistency with the mail-in paper survey (P-2008) conducted during the same time period, the analysis uses only GPS trips recorded in October.

Combining the different I-35W travel surveys into one dataset helps overcome the limitation of small sample size, especially while separating the travelers into groups. While each of the above surveys differed in terms of their focus and the exact wording of the survey questionnaire, all of them contained information on the travelers’ reported travel time for the commute trip. Also the use of two different datasets provided us a larger sample of travelers in the region. The TBI, while older, has a larger sample size and covers all the seven counties in the Twin Cities metropolitan area. On the other hand, the I-35W surveys, while newer, are smaller and are more specific to communities near the I-35W bridge.

Similar to the TBI dataset, the I-35W travel survey dataset is cleaned to remove records with missing or unreasonable attribute values. The final dataset used in this analysis consists of 337 records. Table 2 summarizes the exclusion rules used to obtain this dataset.

**Table 2:** Data exclusion rules - I-35W Travel Surveys.

	No. of observations
Number of initial records in the I-35W Travel Survey dataset	349
Excluding trips with unreasonable reported times	9
Excluding records with unreasonable tau values	3
**Number of records used in the analysis**	**337**

#### Street network

The street network data for the Twin Cities were extracted from the year 2000 Census TIGER/Line files. The Topologically Integrated Geographic Encoding and Referencing (TIGER) files are developed and maintained by the U.S Census Bureau. The TIGER files provide information on various features such as roads, railroads, rivers, as wells as legal and statistical geographic areas [51]. The extracted network was cleaned and stratified into three main categories, arterials, interstates, and local streets, based on the Feature Class Codes (FCC) for the roadway segments provided in the Census TIGER/Line files. The free flow speeds of the road segments provided in the street network were updated with actual speeds to better account for congestion. The average congested speeds in the Twin Cities network were obtained from a GPS study [49], conducted at the University of Minnesota before and after the reopening of the new I-35W bridge.

### Data Preparation

#### Identification of commute route

The first step in the analysis is to identify the route between the commute trip origin and destination for respondents in both datasets, with certain exceptions. For GPS respondents, the raw data obtained from the GPS units provide a complete recording of vehicle trajectories. Each vehicle trajectory recording provides information on the latitude and longitude coordinates, date and time, and the instant speed of the vehicle. The data are used to identify the actual route between the commute trip origin and destination. In order to account for multiple recordings of the same vehicle trajectories, the most frequently used commute route is identified for each traveler, using a previously developed algorithm [2]. The P-2007 and P-2008 respondents provided a rendering of their actual routes on street maps provided for this purpose.

On the other hand, the TBI provides information on the trip origin and destination but does not necessarily identify the actual route chosen by the traveler. Similarly the respondents in the W-2007 survey provided only their home and work locations and did not provide any information on their actual commute routes. Hence the fastest path (computed over roadway segments weighted with average congested speeds derived from the GPS data) between the given trip origin and destination is identified for each trip in these two datasets.

Although travelers will not always follow the fastest route, it is a common route choice criterion for car drivers. The use of the fastest route or shortest travel time route from origin to destination is based on existing research on route choice [52]. As [2] point out, the trip-based modeling paradigm is based on Wardrop’s first principle, in which “the journey times in all routes actually used are equal and less than those which would be experienced by a single vehicle on any unused route” [53]. This assumption has been countered by research on route choice that argue that travel time is not the only criterion that travelers use.

Considering the data available, the shortest travel time path is the best assumption that could be used. Even if the route geometry deviates from the actually chosen route of a survey respondent, it is not expected that the network characteristics in the buffers around these two route alternatives would vary significantly or have a noticeable impact on the role of the network characteristics.

#### Estimation of measures of network structure

The next step is to estimate the trip level measures of network structure along the identified commute route. As mentioned above, the actual route is either obtained directly from the surveys (GPS surveys, P-2007 and P-2008 surveys) or estimated using shortest path algorithms (TBI, W-2007 surveys). A 1-km buffer is created around this actual route and various measures of network structure are estimated within the buffer using the complete street network (including interstates, arterials, and local streets). A similar analysis is carried out using a subnetwork, also called arterial network, consisting of just the interstates and arterials. A 2-km buffer around the actual route is used in the arterial network to estimate select measures of network structure.

A VBA script in ESRI’s ArcGIS 10 was used to calculate the trip level measures of network structure along the commute route. The buffer size, while admittedly arbitrary, provides a geographical definition that is required for the estimation of areal network measures. Various buffer sizes were tested but the final buffer size (1-km/2-km) selection was based on the ability to capture the various network measures and the subsequent performance of these measures in related regression models.

The measures used to quantify network structure within each commute trip buffer are broadly categorized into four main categories: hierarchy, topology, morphology, and scale. It is important to point out that the measures of network structure developed for this analysis are primarily geometric measures based on graph theory. The use of geometric measures was based on data availability and the ease of computation of relevant network measures.

Table 3 provides a summary of the estimated network measures within the trip buffer.

**Table 3:** Estimation of Network Measures.

Network Measures (Unit)	Description	Category	Equation	Notes	Reference
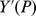 Relative discontinuity,	Captures the differentiation that exists among street networks.	Hierarchy	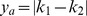 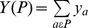 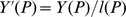	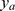 = Discontinuity moving from an upstream link to downstream link, 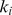 = Hierarchy of the link, 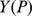 = Discontinuity of the trip along the shortest path, 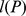 = Total length (km) of trip along the shortest path, 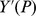 = Relative discontinuity, 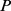 = Shortest path.	[59]
Proportion of limited access roads	Capture the presence of higher hierarchy links, i.e. interstates, within each trip buffer.	Hierarchy	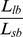	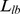 = Length (km) of the limited access roads within the trip buffer, 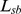 = Total length (km) of the street network within the trip buffer.	
Arterial Treeness*, 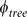	Captures the differences intopology and connection patterns that exist in a real-world street network.	Topology	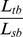	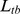 = Length (km) of street segments belonging to a branch network within the trip buffer	[28], [59]
Trip Circuity, 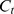	Captures the inefficiencies in the street network from the viewpoint of the traveler. The minimum valueof the 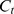 variable is 1.0.	Topology	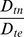	D 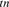 = Network distance (km) between the trip origin and destination, D 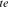 = Euclidean distance (km) between the trip origin and destination.	[60], [61]
Street density, 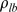 ( 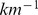 )	Captures the intensity of the street network within the given area.	Scale	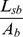	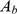 = Area ( 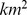 ) of the trip buffer.	
Intersection density, 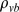	Captures the intensity of the street network within the given area.	Scale	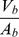	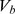 = Number of intersections within the trip buffer.	
P2A, ( 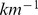 )	Capture the general impedance ofthe street network.	Morphology	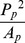	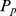 = Perimeter ( 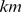 ) of the polygon enclosed by the street network, 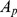 = Area ( 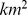 ) of the polygon enclosed by the street network.	

* -Treeness estimated for a subnetwork consisting of interstates and arterials.

#### Estimation of travel time

The next step in the analysis is the estimation of travel time (measured and reported) for the commute trip. The analysis focuses specifically on auto-based (drive alone or carpool) commute trips. The measured travel time is calculated along the identified commute route using the congested speeds in the street network for all respondents (TBI, P-2007, W-2007 and P-2008), with the exception of GPS respondents. For GPS respondents, the time data are directly obtained from the GPS units. The reported travel time is obtained directly from the surveys for GPS survey respondents and W-2007 respondents. The reported travel time is estimated for TBI respondents, P-2007 and P-2008 respondents using the reported trip arrival and departure times. In this analysis, the reported travel time in the surveys is used as a proxy for perceived travel time. A summary of the reported and measured travel time for the two datasets is provided in Tables 4 and 5.

**Table 4:** Summary of reported and measured travel times - TBI, Commute trips.

	All commuters				Commuters that overestimate travel time, *G_o_*				Commuters that underestimate travel time, *G_u_*			
Variable (Unit)	Mean	Std. Dev.	Min	Max	Mean	Std. Dev.	Min	Max	Mean	Std. Dev.	Min	Max
Reported time (min)	24.76	14.40	1.00	90.00	25.76	14.45	1.00	90.00	16.81	11.20	1.00	57.00
Measured time (min)	16.00	10.08	0.51	71.51	15.15	9.29	0.51	61.49	22.76	13.12	2.18	71.51
No. of observations	4,050				3,600				450			

**Table 5:** Summary of reported and measured travel times - I-35W Travel Surveys, Commute Trips.

	All commuters				Commuters that overestimate travel time, *G_o_*				Commuters that underestimate travel time, *G_u_*			
Variable (Unit)	Mean	Std. Dev.	Min	Max	Mean	Std. Dev.	Min	Max	Mean	Std. Dev.	Min	Max
Reported time (min)	27.48	14.72	1.00	90.00	28.33	15.15	4.00	90.00	22.40	10.61	1.00	40.00
Measured time (min)	18.62	10.31	0.65	47.72	17.13	9.21	0.65	47.48	27.58	12.01	5.73	47.72
No. of observations	337				289				48			

**Figure 1:**
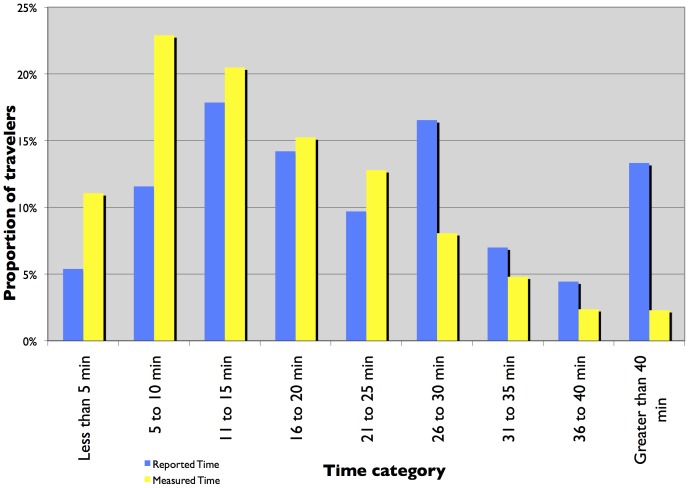
Frequency plot of reported and measured commute time – TBI.

**Figure 2:**
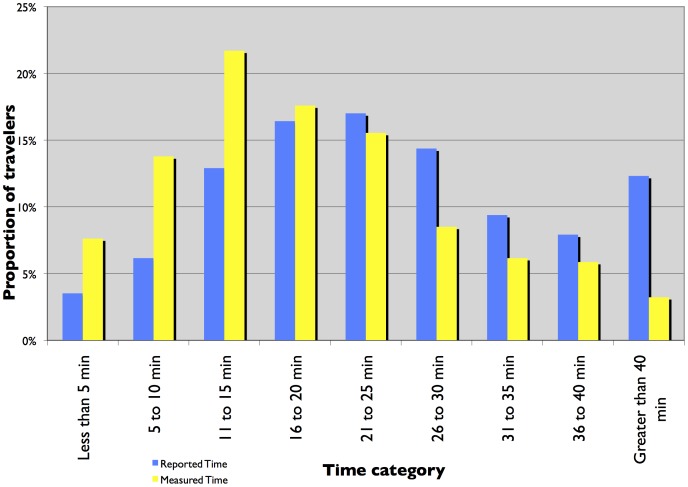
Frequency plot of reported and measured commute time - I-35 W surveys.

**Figure 3:**
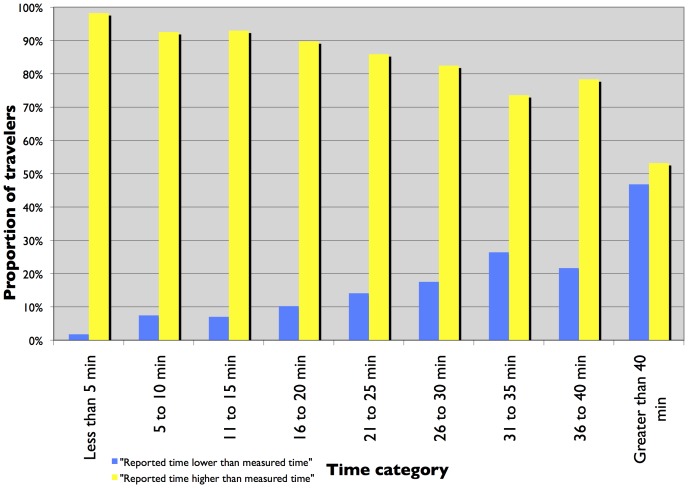
Frequency plot of the ratio of reported to measured commute time, stratified by measured time – TBI.

**Figure 4:**
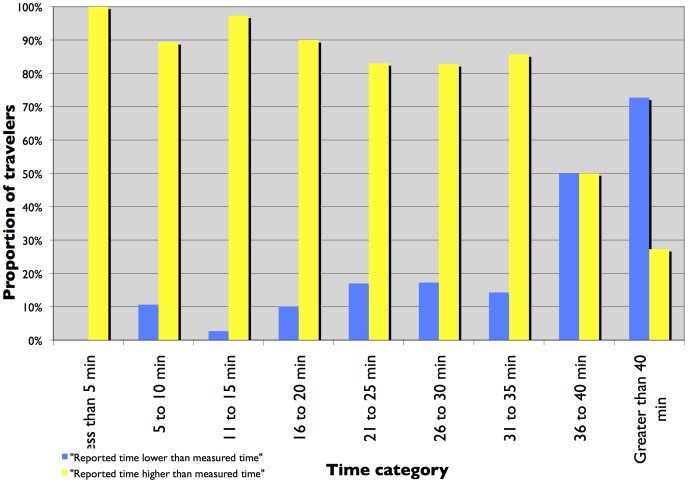
Frequency plot of the ratio of reported to measured commute time, stratified by measured time - I-35 W surveys.

Figures 1 and 2 present a histogram plot of reported and measured travel times from TBI and the I-35 W surveys. The histograms show a change in trend at the 26 to 30 minute time category. For time categories under 25 minutes, the proportion of travelers is higher for measured commute time than reported commute time. For time periods greater than 25 minutes, the proportion of travelers is higher for reported commute time than measured commute time. At first glance, it seems that for longer trips, travelers report the travel time to be higher than for shorter trips. But closer investigation using histogram plots of the ratio of reported time to measured time, stratified by measured time, presented in Figures 3 and 4 show that the proportion of travelers that overestimate their travel time (reported time is greater than measured time) is highest for shorter trips and reduces as the trip duration increases. The trend is clearer for travelers in the TBI compared to the I-35W surveys but the pattern is consistent between the two datasets. This is consistent with Vierordt’s Law which states that within a series of stimulus intervals, longer intervals tend to be underestimated while shorter intervals tend to be overestimated [54].

### Analysis

This section describes the analyses conducted using the travel survey and street network data. The first analysis is a statistical t-test comparison of network measures between two groups of commuters, namely overestimating commuters and underestimating commuters. The second analysis is a linear regression model predicting the ratio of perceived travel time and measured travel time, using the network measures as independent variables. The t-test comparison analyzes the individual influence of each network measure while the regression analysis considers the combined influence of the network measures.

### T-test Analysis

#### Comparison of under and over-estimating commuters

The ratio of perceived travel time and measured travel time is calculated for each respondent in both datasets.
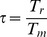
(1)where,


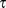
 = Ratio of perceived (reported) travel time to measured travel time,


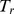
 = Perceived (reported) commute travel time, in minutes,


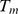
 = Measured commute travel time, in minutes.

Travelers are then classified into two groups based on this ratio.

- Overestimating Group, 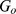 : Travelers perceive their commute travel time to be higher than it actually is,

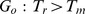


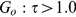
(2)

- Underestimating Group, 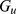: Travelers perceive their commute travel time to be lower than it actually is

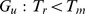


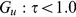
(3)


A simple comparison of the reported and measured travel time for the two traveler groups is provided in Tables 4 and 5. The comparison highlights the differences between the two traveler groups (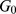, 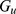). Looking at the TBI dataset presented in Table 4, we see the mean reported travel time is higher for the travelers in 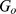 group compared to the 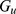 group. On the other hand, the mean measured travel time is lower for the travelers in the 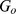 traveler group compared to the 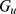 group. This pattern is seen in the I-35W travel survey dataset as well.

This step in the analysis compares the trip-level measures of network structure between the two traveler groups, using the statistical t-test. The t-test checks if the mean value of a specific network measure is statistically different between the two traveler groups. The t-test comparison is conducted separately for each network measure in both datasets.
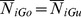
(4)



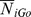
 = Mean of network measure, *i*, in the overestimating group, 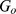,


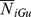
 = Mean of network measure, *i*, in the underestimating group 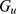.

#### Hypotheses

The hypotheses formulated for the t-test analysis are presented below:

Aspects of network structure (operational variables: 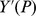) that increase travel complexity will increase perceived travel time (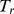). Hence,

- H1 - The mean of 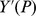 is higher for travelers in the overestimating group (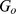) compared to travelers in the underestimating group (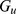).

Aspects of network structure that increase network speed (operational variables: proportion of limited access roads) will decrease perceived travel time (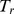). Therefore,

- H2 - The mean of the proportion of limited access roads is lower for travelers in the overestimating group (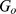) compared to travelers in the underestimating group (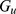).

Aspects of network structure that decrease network speed (operational variables: 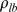, 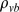) will increase perceived travel time (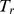). Thus,

- H3 - The mean of 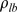 is higher for travelers in the overestimating group (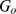) compared to travelers in the underestimating group (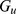).
- H4 - The mean of 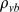 is higher for travelers in the overestimating group (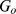) compared to travelers in the underestimating group (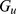).

Aspects of network structure that increase network travel distance between fixed origins and destinations (operational variables: 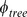, 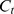, P2A) will increase perceived travel time (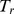). Hence,

- H5 - The mean of 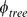 is higher for travelers in the overestimating group (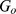) compared to travelers in the underestimating group (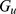).
- H6 - The mean of 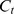 is higher for travelers in the overestimating group (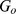) compared to travelers in the underestimating group (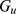).
- H7 - The mean of P2A ratio is higher for travelers in the overestimating group (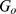) compared to travelers in the underestimating group (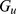).

### Regression Analysis

#### Predicting the ratio of travel time

The above section presented individual t-test comparisons of network measures between the two traveler groups. Here, the independent network measures are combined in regression models that predict the ratio (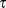) of perceived travel time (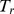) to measured travel time (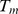). Network structure is complex and the measures estimated here capture certain aspects of the structure. How these measures interact with each other is not easily understood. The t-test comparisons identify how the measures perform individually while the regression model looks at the combined effects of these measures.

The TBI dataset is used to estimate the regression model due to the sample size. A correlation analysis of the estimated network measures is conducted to ensure that only independent variables are included in the regression model. Based on the analysis, the intersection density variable is dropped from the analysis due to its high correlation with the street density variable.

The functional form of the regression model is given below:
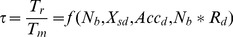
(5)where,


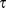
 = Ratio of perceived (reported) travel time to measured travel time,


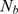
 = Measures of street network structure within the trip buffer,


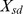
 = Socio-demographic characteristics (e.g. age, household size, household income),


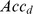
 = Distance based measure of accessibility, estimated as the straight line distance between the traveler’s residence and the downtowns of Minneapolis and St. Paul,


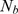
*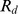 = Interaction of each measure of network structure with route distance.

The route distance is calculated from the identified commute route of each respondent. Two regression models are estimated using the above functional form.

#### Model 1

The first model (Model 1) uses a reduced version of the above functional form. This model predicts the ratio of travel time as a function of just 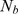, 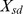 and 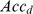 variables. The interaction of each network measure with route distance (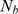
*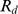) is not included as an independent variable in the analysis.

#### Model 2

This model uses the complete function form provided in equation 5. It includes the interaction terms (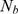
*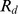) in addition to the 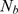, 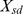 and 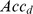 variables. In order to avoid collinearity between the variables, the network measures and the route distance variable are standardized before estimating the interaction terms. A standardized variable is one that has been rescaled to a mean of 0 and a standard deviation of 1 [55]. The standardized interaction terms are included as independent variables in the regression model predicting the ratio of reported and measured travel time.

The use of the two models is to better understand the influence of network measures and to ensure that the performance of the network measures is not dependent on the actual route length. The results from the two models are described in the following section.

## Results

### T-test Analysis

#### Difference in network structure between under- and over-estimating commuters

The results of the t-test analysis for both datasets are presented in Table 6. The results show that most measures of network structure differ significantly between the two traveler groups. The measures of relative discontinuity, street density and intersection density perform as hypothesized. These measures have a higher mean for travelers in the overestimating group (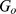) and are statistically different compared to travelers in the underestimating group (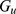). The proportion of limited access roads has a lower mean for travelers in the overestimating group (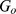) and is statistically different compared to travelers in the underestimating group (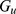). This is also in line with the hypothesis but is however seen only in the I-35W travel surveys. The mean of the arterial treeness measure is statistically different and is lower for travelers in the overestimating group (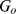) compared to the underestimating group (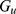) in the TBI dataset, contradicting the hypothesis. It is however is not significant in the I-35W surveys. The mean of the P2A variable and the measure of trip circuity are lower for travelers in the overestimating group (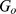) compared to travelers in the underestimating group (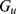) but is statistically significant only in the TBI dataset.

**Table 6:** T-test comparisons of estimated measures of network structure.

Ho: Difference between means is zero								
	TBI, Commute trips				I-35W Travel Surveys, Commute Trips			
Network Variables	Mean( 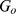 )	Mean( 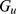 )	t	Sig	Mean( 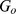 )	Mean( 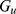 )	t	Sig
Relative discontinuity, 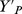	0.298	0.199	6.796	***	0.340	0.255	2.126	**
Proportion of limited access roads	0.055	0.054	0.811		0.081	0.103	−2.970	***
Arterial Treeness, 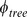	0.009	0.011	−2.043	**	0.013	0.012	0.370	
Trip Circuity, 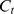	1.333	1.365	−2.293	**	1.392	1.383	0.127	
P2A	24.513	24.906	−2.617	***	23.097	22.873	0.693	
Street density, 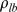	18.275	15.164	12.627	***	19.426	15.617	4.913	***
Intersection density, 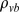	28.885	22.374	11.811	***	40.901	36.133	2.907	***
Number of observations	3,600	450			289	48		
Total observations	4,050				337			

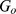
: Travelers perceive their commute travel time to be higher than measured travel time.

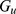
: Travelers perceive their commute travel time to be lower than measured travel time.

* p<.10,

** p<0.05,

*** p<.01.

The two datasets show slight differences in the influence of network measures. The differences could be attributed to the differences in the data collection, the time period of the travel surveys and the methodology used to obtain the actual commute route and travel time information. However the results are mostly in line with the hypotheses and show that network measures do vary between traveler groups.

The results presented in Table 6 are from the t-test analysis for both datasets, using all data records. An additional t-test comparison is conducted by stratifying the data for varying route length/distance. This is done to better understand the behavior of the network variables and to ensure that difference between the two travelers groups (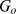, 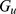) is not due to the actual route length.

As explained before, the route distance is calculated from the identified commute route for each respondent. The TBI and I-35W survey datasets are each subdivided into three groups based on the route distance, namely, route distance less than 20 km, route distance between 20 km and 40 km and route distance greater than 40 km. The t-test analysis is then repeated for each of these subgroups. The results, not presented here, show patterns of the influence consistent with the t-test analysis presented in Table 6.

The next section details the results of regression models estimated using the above mentioned network measures.

### Regression Analysis

#### Predicting the ratio of travel time

The results of the two linear regression models (Models 1 and 2) and the associated elasticity estimates are presented in Table 7 and 8. Table 7 shows the results of the two regression using all the commuters in the TBI dataset. Table 8 shows the results of the two regression models using just the travelers that over perceive (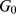) their commuter travel time. Both models include non-network variables as control variables to see if the influence of network measures exist even in the presence of these variables. The results from Model 1 and 2 show that the network variables influence the ratio (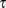) of reported travel time to measured travel time. For example, refer to Table 7. The relative discontinuity variable is positive and highly significant in both models, confirming our hypothesis. Similarly the street density is positive and significant in both models. This corroborates the hypothesis that the higher street density in the network leads travelers to perceive their travel time to be higher resulting in an increase in the ratio (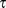). The arterial treeness variable is also positive and significant, as hypothesized. The proportion of limited access roads is negative and highly significant in both models as expected. The P2A variable is positive in both models but is however significant only in Model 2. The trip circuity variable is positive in both models contradicting our hypothesis but is not significant in either models.

**Table 7:** Predicting the ratio of reported travel time to measured travel time - TBI, Using all commuters.

Dependent variable, 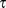 = Reported travel time (*T_r_*)/Measured travel time (*T_m_*)									
		Model 1				Model 2			
Independent Variables (Unit)	Hypothesis	Coef.	Sig	t	Elasticity	Coef.	Sig	t	Elasticity
Distance to downtown Minneapolis ( 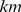 )		−0.008	***	−2.882	−0.073	−0.006	**	−2.207	−0.054
Distance to downtown St. Paul ( 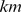 )		0.003		1.293	0.035	0.002		1.244	0.023
Relative discontinuity, 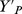 ( 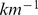 )	+S	0.972	***	8.805	0.152	1.384	***	18.128	0.217
Proportion of limited access roads	−S	−2.032	***	−4.040	−0.061	−1.996	***	−4.510	−0.060
Arterial Treeness, 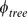	+S	2.239	*	1.958	0.011	1.740	*	1.801	0.009
Trip Circuity, *C_t_*	+S	−0.096		−1.189	−0.070	−0.055		−0.943	−0.040
P2A ( 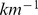 )		0.006		0.681	0.080	0.014	*	1.946	0.188
Street density, 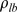 ( 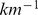 )	+S	0.038	***	7.087	0.372	0.032	***	7.020	0.313
Relative discontinuity*Shortest Path route distance		NA	NA	NA	NA	0.138	***	4.171	−0.032
Proportion of limited access roads*Shortest Path route distance		NA	NA	NA	NA	0.001		0.077	0.000
Arterial Treeness*Shortest Path route distance		NA	NA	NA	NA	−0.029	*	−1.920	0.000
Trip Circuity*Shortest Path route distance		NA	NA	NA	NA	0.007		0.360	0.000
P2A*Shortest Path route distance		NA	NA	NA	NA	0.062	**	2.193	0.003
Street density*Shortest Path route distance		NA	NA	NA	NA	−0.056	**	−2.568	0.009
Constant		0.983	***	3.004		0.754	***	2.844	
Number of observations		4,050				4,050			
R-squared		0.152				0.162			
Adj. R-squared		0.149				0.157			

* p<.10,

** p<0.05,

*** p<.01.

**Table 8:** Predicting the ratio of reported travel time to measured travel time, controlling for route distance - TBI, using only commuters that over estimate travel time (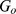).

Dependent variable, 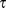 = Reported travel time (*T_r_*)/Measured travel time (*T_m_*)									
		Model 1				Model 2			
Independent Variables (Unit)	Hypothesis	Coef.	Sig	t	Elasticity	Coef.	Sig	t	Elasticity
Distance to downtown Minneapolis ( 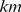 )		−0.007	***	−2.598	−0.057	−0.007	**	−2.250	−0.057
Distance to downtown St. Paul ( 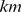 )		0.002		0.973	0.021	0.002		0.930	0.021
Relative discontinuity, 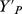 ( 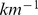 )	+S	0.937	***	8.135	0.142	1.263	***	16.016	0.191
Proportion of limited access roads	−S	−2.057	***	−3.830	−0.058	−2.095	***	−4.740	−0.059
Arterial Treeness, 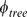	+S	2.895	**	2.141	0.013	2.013	*	1.720	0.009
Trip Circuity, 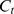	+S	−0.037		−0.303	−0.025	0.120	*	1.762	0.081
P2A ( 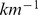 )		0.003		0.299	0.037	0.009		1.239	0.112
Street density, 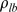 ( 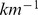 )	+S	0.031	***	5.319	0.288	0.023	***	5.004	0.214
Relative discontinuity*Shortest Path route distance		NA	NA	NA	NA	0.107	***	2.899	−0.023
Proportion of limited access roads*Shortest Path route distance		NA	NA	NA	NA	0.002		0.111	0.000
Arterial Treeness*Shortest Path route distance		NA	NA	NA	NA	−0.030	*	−1.650	0.000
Trip Circuity*Shortest Path route distance		NA	NA	NA	NA	0.042		1.395	−0.003
P2A*Shortest Path route distance		NA	NA	NA	NA	0.041		1.292	0.002
Street density*Shortest Path route distance		NA	NA	NA	NA	−0.083	***	−3.086	0.011
Constant		1.185	***	3.240	NA	0.912	***	3.341	NA
Number of observations		3,600				3,600			
R-squared		0.139				0.148			
Adj. R-squared		0.136				0.143			

* p<.10,

** p<0.05,

*** p<.01.

The consistency of results in both Model 1 and 2, shown in Table 7, confirm that the influence of network measure is independent of the actual route distance. The inclusion of the route distance via the interaction terms in Model 2 does not change the influence of the network measures and only results in a slight increase in the adjusted 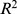. A similar influence of network variables on the ratio of travel time (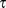) is seen in Table 8 as well.

The socio-demographic variables act as control variables in this analysis and are hence not elaborated here for brevity. The results confirm a relation between the street network structure and individual perception of travel time, after controlling for non-network variables.

## Discussion

The objective in this paper is to identify differences in how travelers perceive their commute travel time. Another objective is to relate these differences in perception to the underlying measures of network structure along the commute route. To that effect, travelers are categorized into two groups, based on the ratio of their reported travel time and measured travel time. Statistical t-test comparisons are conducted to identify differences in individual network measures between the two groups followed by regression models estimated to analyze the combined effect of these measures. The t-test analyses presented here identified statistically significant differences between the two traveler groups and the regression models confirmed the same.

An understanding of how travelers perceive their travel time is important due to its effect on actual travel. Recent research efforts have confirmed this relation between street network structure and observed (actual) travel at the individual, household and metropolitan level [56]. This paper focuses on the underlying theory, specifically on why network structure influences actual travel. The hypothesis presented here is that network design influences traveler perceptions, more specifically the perceptions of distance/travel time. This perception of distance/travel time in turn influences the actual travel by affecting choice of destination, mode, route, and whether to engage in activities.

There are many factors that affect travel in a region. The simplified models developed here imply that network design should be one of the tools to be considered in analyzing travel. The results presented here along with related analyses [56] complement the role of conventional measures of urban form and the built environment. This understanding and application of network structure measures to network design is critical in the design of new networks, especially in developing countries, and enhancements to existing systems.

The elasticity estimates from the two linear regression models are presented in both Table 7 and 8. These estimates highlight the change in the ratio of travel time, 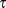, due to an unit change in the independent network variable, considering everything else to be the same. For example, refer again to Table 7. A unit increase in the relative discontinuity between a commute trip origin and destination results in a 0.15–0.22 decrease in 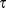. Similarly a unit increase in the street density results in a 0.31–0.37 increase in 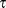 for the commute trip. The elasticity estimates provide a magnitude of influence of the independent network variables.

The analyses presented here do have some limitations. The data analyzed here are compiled from different sources. The compilation helped overcome limitations in sample size. However the actual wording and format of the survey questionnaire differed between the various surveys. This resulted in minor differences in how the perceived and actual route/travel time information was obtained from travelers. The reported travel time is either obtained directly (GPS survey, W-2007) or estimated from reported arrival and departure times (TBI, P-2007 and P-2008). Similarly the actual route information is either obtained directly (GPS, P-2007 and P-2008) or identified using shortest path algorithms (TBI, W-2007). Future extensions would be to collect relevant survey data on commute routes and travel time.

The analysis currently focuses on auto based commute trips and the underlying street network structure in the analysis of the perception of travel time. Research on transit network design shows that key components of network design have a significant impact on ridership and transit system performance [57], [58]. Thus to obtain a comprehensive understanding of travel, it is important to consider other transportation modes and their networks.

The measures of network structure used in this analysis are graph theory based geometric measures that capture certain aspects of network structure and design. While the geometric representation captures the underlying structure or connectivity in a street network, the use of a true topological approach, which not only considers each individual element of the network but also takes into the account inter-relationships between adjacent network elements, may provide a higher level of abstraction and variation [57]. Future research could therefore try to relate topological measures of network structure with travelers perception of travel time.
